# Pseudologia Fantastica in the Emergency Department: A Case Report and Review of the Literature

**DOI:** 10.1155/2017/8961256

**Published:** 2017-05-10

**Authors:** Robyn Thom, Polina Teslyar, Rohn Friedman

**Affiliations:** Beth Israel Deaconess Medical Center, 330 Brookline Ave, Boston, MA, USA

## Abstract

Psychiatrists commonly encounter deception in the emergency department. This article presents the case of a patient who presents to the emergency department with an unusual and elaborate web of deceptions along multiple themes including feigning medical illness, multiple losses, and grandiose academic and athletic achievements. We review the clinical characteristics of pseudologia fantastica and discuss how this patient's constellation of malingering, factitious disorder, and personality disorder suggests this diagnosis.

## 1. Introduction

Pseudologia fantastica, a fascinating psychiatric syndrome where patients represent certain fantasies as real occurrences, has received little attention in the English literature: there are fewer than thirty case reports in the English literature. Pseudologia fantastica goes by multiple different names including pathologic lying and mythomania. In this case report and discussion, we begin by providing a case that illustrates pseudologia fantastica and then discuss the phenomenologic, nosologic, psychodynamic, and treatment issues of this construct.

## 2. Case

The patient is a 28-year-old man with a history of major depressive disorder, hepatitis C, biliary colic (status postcomplicated cholecystectomy), multiple concussions, and chronic back pain who presented to the emergency department with abdominal pain clinically concerning for acute appendicitis. He localized his pain to the right lower quadrant, complained of right lower quadrant pain with palpation of the left lower quadrant (Rovsing's sign), and indicated his pain was reproducible with extension of the right hip (psoas sign). His vital signs on presentation were notable for the absence of fever or tachycardia and his initial laboratory findings revealed normal chemistries and a normal white blood cell count of 4.2. He received intravenous morphine and an urgent CT scan of his abdomen. Enroute to the CT scanner, the patient mentioned that he had been struggling with sadness and suicidality since his pregnant fiancée had recently been killed in an automobile accident. The emergency physicians felt the pretest probability for acute appendicitis was extremely high and asked the psychiatry consult-liaison team to “evaluate him quickly before he goes to surgery.” Ultimately, the imaging was negative for acute appendicitis, surgery was cancelled, and his disposition from the emergency department was left to psychiatry.

Review of the electronic medical record showed that the patient had established care at this institution one month prior to this presentation when he presented with biliary colic resulting in a lengthy hospitalization for a complicated cholecystectomy, challenging postoperative pain management, and a new diagnosis of hepatitis C. During that hospitalization, he was seen by social work who documented that he was baffled as to how he had contracted hepatitis C but offered that he had endured many losses in his life including the death of his mother when he was 6 years of age and the death of his brother when he was 19, so felt he had developed strong coping skills. In terms of his social history, he reported that he was engaged to be married and worked as a mathematics and physics professor at a prestigious university as well as an engineering consultant in the private sector. He also reported that he had sustained a number of musculoskeletal injuries resulting in chronic pain while playing Division I football in college and that he had been drafted by the National Football League. Upon discharge from that admission, he established care with a primary care physician for ongoing management of chronic pain. At his initial appointment he reiterated the social history he provided to the social worker and signed a narcotics agreement.

On initial assessment in the emergency department by the psychiatry consult-liaison team, the patient was observed to be a young, overweight Asian male dressed in a plain white T-shirt and track pants. His hair was greasy, his fingernails were long and dirty, and one of the lenses of his eyeglasses had a small crack. He made poor eye contact and focused his gaze on his tablet computer for most of the interview. His affect was withdrawn and had minimal range. On interview, he began the conversation by requesting placement at an inpatient psychiatric facility for electroconvulsive therapy, reporting that he had been suffering from very low mood since his pregnant fiancée had been killed by a drunk driver eight months ago. Because the electronic medical record indicated that he had been engaged to be married just one month ago, we asked him to confirm the date of his fiancée's death, which he could not recall. He answered all questions in a matter-of-fact tone and showed very little range of affect. In terms of his mood, he did not elaborate on his experience other than to say he felt “depressed and suicidal” with the vague plan of jumping in front of a train. He evaded further discussion of his mood symptoms by spontaneously offering details of his social history. He spoke about his profession as a tenured mathematics and physics professor at a prestigious university although when asked about the nature of his research he could only vaguely describe studying “time bends in space using some of Einstein's old formulae.” He spoke in some detail about his career as a varsity football player, citing football injuries as the source of chronic back pain. When asked to provide a collateral contact, he reported that both of his parents, multiple siblings, and cousins had died during his early childhood.

The patient was accompanied by a male friend who was casually but neatly dressed and of approximately the same age. The patient provided us with permission to speak with his friend who appeared uncomfortable, saying “I didn't know I'd have to talk!” He reported that he and the patient were work colleagues who had known each other for a few years but refused to reveal where they worked. He provided no further information, other than saying “He's been really depressed and I just know he needs help before something happens.” He then quickly left the hospital without saying goodbye to the patient.

Although, the patient initially reported that both parents were deceased, his father was listed as an emergency contact in his medical record. When confronted with this, the patient said that this was his step-father and gave consent for contact. The father reported that, five years ago, his son graduated with poor grades from a university which does not have a Division I football team. He confirmed the patient had played football in high school and had sustained multiple concussions. The year after graduation, the patient had lived with his parents briefly, but because of escalating narcotic use and lack of employment was asked to leave. Since then, he has suffered from severe opioid use disorder and has been homeless and unemployed. He has travelled to various hospitals within the city and even out of state to seek pain medication and care and has told a similarly fictional narrative to other physicians.

We obtained information from a local emergency community outreach agency which indicated that the patient had presented with the chief complaint of suicidality to multiple emergency departments in the city resulting in two previous inpatient psychiatric stays over the past year. We obtained records from his most recent inpatient psychiatric hospitalization about six months ago, where he presented with depression related to his girlfriend's putative recent breast cancer diagnosis. He was discharged on an antidepressant, a mood stabilizer, and oxycodone for chronic back pain.

When gently confronted with these inconsistencies, the patient appeared unperturbed and easily provided further elaborate details to explain them. However when further pressed, he stated he believed he needed a “dramatic” reason for his depression and suicidality to receive help and asked, “Can I just be depressed and suicidal?” He appeared perplexed as to why we attempted to clarify his previous statements or their relevance for his care. Despite this, he continued to state that he felt very depressed and would not be able to maintain his safety in the community.

## 3. Discussion

### 3.1. The Phenomenology of Pseudologia Fantastica

The term pseudologia fantastica was first coined by the German physician, Anton Delbrueck, in 1891 to describe the phenomenology of a group patients who told lies that were obviously extreme and fantastical with a clear departure from reality to the observer, yet perceived by the patients themselves as within the realm of possibility [1]. Delbrueck described a case series of five patients whose tales could not be solely attributed to ordinary lying, false memory, or delusions. He coined the term “pseudologia fantastica” to describe patients who tell complex tales where delusions seemed to coexist with lies [2]. Since then, pseudologia fantastica has undergone subsequent further study, and while there is no current gold-standard definition of this entity, subsequent reviews [3–7] have identified key characteristics, including the following:

1. Chronic lying/storytelling that is unrelated to or out of proportion to any clear objective benefit;
2. Qualitatively the stories are dramatic, detailed, complicated, colorful, and fantastic;
3. The stories typically feature the pseudologue as the hero or victim and seem geared to achieve acceptance, admiration, and sympathy;
4. In terms of insight, the pseudologue lies somewhere along a spectrum between conscious deceit and delusion, not always conscious of his motives and seeming at least intermittently to believe his stories yet never to reach the level of conviction that would indicate a loss of reality-testing.

Reviews of pseudologia fantastica indicate that the pseudologue is typically of normal intellect but of superior verbal ability. 40% have a history of a central nervous system abnormality such as epilepsy, abnormal electroencephalogram, head trauma, or central nervous system infection. At least 50% demonstrate a history of peregrination among different healthcare institutions, and 25% simulate a physical illness [3].

### 3.2. Nosology of Pseudologia Fantastica in the DSM 5

Although pseudologia fantastica is not coded in the DSM 5, it has historically been associated with factitious disorder. Factitious disorder imposed on self (frequently referred to as Munchausen syndrome) is defined as the falsification of physical or psychological signs or symptoms and the presentation of self as ill, impaired, or injured in the absence of obvious external rewards [8]. In cases in which the tales all relate to presenting himself as ill, impaired, or injured, factitious disorder might apply. However, the pseudologue typically tells tales on a wide range of themes, some of which do not have any relation to feigned physical or psychological signs or symptoms. Moreover, sometimes there may be obvious external rewards, such as getting shelter or medications; but the stories are out of proportion to any obvious external rewards.

Pseudologia fantastica has sometimes been equated with malingering. Malingering, now a V-code in DSM 5, is defined as “the intentional production of false or grossly exaggerated physical or psychological symptoms, motivated by external incentives such as avoiding military duty, avoiding work, obtaining financial compensation, evading criminal prosecution, or obtaining drugs.” [8] In general, malingering is seen as instrumental, calculated to a goal, and “adaptive” in that sense. Pseudologic tales are often qualitatively far too dramatic and colorful to be considered adaptive to acquire an external incentive. In fact, the level of dramatism and clear departure from reality actually lead to discovery of the falsification. This tests the concept of “adaptiveness” and suggests that something else is going on. In addition, as with factitious disorder, malingering in DSM 5 focuses on physical or psychological symptoms. Lies or stories that were not about symptoms would not be covered.

Delusional disorder or other psychotic disorders might be in the differential if a patient has full conviction in an unreal story; however, the stories are nonbizarre, the patient's thought process is well-organized, and the tales do not reach the level of conviction required to be considered a delusion. In this patient's case, when confronted he was able to relinquish his tale, further differentiating this from a delusion.

In addition, cases consistent with the phenomenology of pseudologia fantastica have been described with comorbid substance use disorder [9], developmental delay [10], gender disturbance [11], posttraumatic stress disorder [12], mood disorder [6], and personality disorder [6, 13–15]. Patients who present with pseudologia fantastica may additionally present with borderline, antisocial, or other personality disorder traits. In the case of this patient., he demonstrated several narcissistic personality traits. Many of his stories were on the themes of grandiose professional, academic, and athletic achievement, highlighting the need for excessive admiration and preoccupation with a fantasy of unlimited success and power. His inability to understand why his providers in the emergency department were attempting to address the lack of veracity of his stories, as well as his goal to obtain inpatient hospitalization and narcotic medications, demonstrate a lack of empathy and an interpersonally exploitative quality. Thus, in addition to his stories being characterized as pseudologia fantastica, the patient also met diagnostic criteria for a substance use disorder and likely narcissistic personality disorder.

In summary, the chronic pattern of storytelling seen in pseudologia fantastica cannot be wholly accounted for by any single standard DSM 5 diagnosis and, in fact, may present in the context of multiple DSM 5 diagnoses. We propose that pseudologia fantastica be viewed as a construct used to describe a particular pattern of storytelling that crosses but does not neatly fit into multiple diagnostic categories, including the somatic symptom and related disorders (factitious disorder), malingering, and psychotic disorders (delusional disorder). This notion is reminiscent of Delbrueck's initial description of pseudologia fantastica as the mixture and coexistence of lies and delusions [2]. Finally, patients presenting with pseudologia fantastica should be carefully evaluated for comorbid psychiatric diagnoses including substance use disorders, mood disorders, and personality disorders.

### 3.3. Psychodynamic Understanding of Pseudologia Fantastica

In reviewing the case report literature on the subject [4, 6, 7, 9–15], we identified a compensatory enhancing of self-esteem in the face of shame as a common qualitative theme among many of the stories. For example, Teaford et al. described a 14-year-old girl with pervasive developmental delay who began making up self-aggrandizing stories of academic success and intelligence when she became aware she was lagging behind her peers academically [10], while Snyder described a case of an 18-year-old man who told stories about being a well-connected drug dealer in response to feelings of inadequacy when compared to his brothers [6]. From a psychodynamic perspective, pseudologic tales may be conceptualized as manifestations of a constellation of primitive defenses against painful affects that may be seen in a range of psychiatric disorders. Not only do pseudologues reject reality, but they also actively create a new reality that allows for wish fulfillment. Dissociation, temporary but drastic modification of one's personal identity to relieve distress, is also a hallmark defense of pseudologia fantastica. The pseudologue moves between conscious deceit and delusion, at times believing his tales while at others able to acknowledge their mendacity, thereby demonstrating a dissociative quality. As summarized by Deutsch, the psychic function of storytelling in pseudologia fantastica is not focused on achieving goal-directed external gain, but rather on the gratification inherently tied to the communication of the story itself. Communicating pseudologic daydreams as if they were reality serves the function of relieving the pseudologue of the obligations of real life [16].

### 3.4. Treatment

With few case studies and no clinical research on this subject, the optimal management strategy for pseudologues remains controversial and unclear. Two possible avenues of interaction have been described, with the first being confronting the pseudologue with his deceptions, and the second being showing disinterest in the tales but maintaining interest in the patient himself. Both Teaford et al. and Hoyer describe greater success with the latter strategy and in fact found that confrontation resulted in increased frequency of pseudologic output [10, 17].

The “lying” patient often evokes frustration and resentment in his providers [6], which may lead clinicians to a punitive prosecutorial type of confrontation which may only lead the patient to go elsewhere with the same behavior rather than changing the behavior. In the literature on factitious disorder, a similar problem led Hollender and Hersh to recommend having the primary care physician confront the patient so the psychiatrist can avoid the prosecutorial role [18]. Similarly, Eisendrath proposed an “inexact interpretation,” where the interpretation is partially correct but incomplete, directed at the underlying dynamics without directly confronting the false symptoms. His premise behind “inexact interpretation” is that factitious behaviors serve an important psychologic function and that a patient can only recover from them if they feel safe. In the same vein, an interpretation that captures the underlying dynamics of the pseudologue without directly confronting the stories is more likely to be successful than a prosecutorial approach [19].

## 4. Outcome

We were confronted with the challenge of determining the patient's disposition from the emergency room in the face of his elaborate deception. As we have noted, recognition of pseudologia does not preclude underlying psychiatric disorders. Even after acknowledging his previous deceptions, the patient staunchly stood by his suicidality. Perhaps the patient could only seek help for his “true” statement of helplessness, depression, and suicidality by intertwining it with “lies” and self-aggrandizing fantasies. Ultimately, the decision was made to hospitalize the patient on a dual-diagnosis inpatient unit to achieve opioid detoxification and diagnostic clarification and to attempt to engage him in treatment beyond what was possible in the emergency department setting.

## 5. Conclusion

Pseudologia fantastica is a rare but dramatic clinical psychiatric presentation. Performing a safety assessment and developing a therapeutic alliance can be challenging with these patients, since deception easily engenders resentment among treatment providers. Labeling a case as pseudologia fantastica may be a punitive expression of that resentment and lead to overlooking underlying psychiatric disorders which must be accurately diagnosed and treated. Reframing a pseudologue from a “liar” to a patient with impaired self-esteem whose primitive defense mechanisms create a fluid ability to move between reality and fantasy (akin to what has been called “pseudo-lying” in children, a murky area between fully conscious lying and daydreaming that is typical for children) [17] may increase our ability to establish rapport with and treat these challenging patients.
